# Management of febrile neutropenia in the United Kingdom: time for a national trial?

**DOI:** 10.1038/sj.bjc.6602872

**Published:** 2005-12-06

**Authors:** H Innes, L Billingham, C Gaunt, N Steven, E Marshall

**Affiliations:** 1 Clatterbridge Centre for Oncology, Bebington, Wirral, Merseyside CH63 4JY, UK; 2 Cancer Research UK Institute for Cancer Studies, University of Birmingham, Birmingham, UK

**Keywords:** febrile neutropenia, low-risk, oral antibiotics, UK practice

## Abstract

Recent advances in febrile neutropenia (FN) have highlighted the value of risk stratification and the evolving role of oral antibiotics with early hospital discharge in low-risk patients. The aim of this study was to survey whether these advances have been translated into routine clinical practice in the UK. Questionnaires were sent to cancer clinicians across the UK to determine clinicians' routine management of FN, including use of risk stratification, antibiotic regimen and criteria for hospital discharge. In all, 128 clinicians responded, representing 50 cancer departments (83%). Only 38% of respondents stratify patients according to risk and with substantial variation in the criteria defining ‘low-risk’. Furthermore, only 22% of clinicians use oral antibiotics as first-line treatment in any patients with FN, but this was significantly greater among clinicians who do compared to those who do not stratify patients by risk, 51 *vs* 4% (*P*<0.0001). These findings suggest a slow and/or cautious introduction of newer strategies for the management of low-risk FN in the UK. However, 84% of respondents confirmed their willingness to participate in a trial of oral antibiotics combined with early discharge in low-risk FN.

For more than 30 years the standard management for all patients developing febrile episodes while neutropenic has been in-patient treatment with broad spectrum intravenous (i.v.) antibiotics (Hughes *et al*, 1990; Bodey *et al*, 1997; Hughes *et al*, 1997). However, recent evidence has prompted a re-assessment of febrile neutropenia (FN), with increasing realisation that such intensive treatment may not be necessary or appropriate for all patients. This viewpoint has stemmed from the recognition that FN is not a single entity but rather represents a spectrum of severity. Only a small proportion of patients with FN will develop serious medical complications (16% overall, 12% of patients with solid tumours/lymphomas and 18% of patients with haematological malignancies) and fewer than 5% will die as a result of the episode (4.8%; Klastersky *et al*, 2000). Thus a majority of episodes of FN may be described as being ‘low-risk’. Several groups of investigators have independently developed prognostic indices in FN in an attempt to identify criteria by which to define ‘low-risk’ (Rubin *et al*, 1988; Talcott *et al*, 1992; Viscoli *et al*, 1994; Elting *et al*, 1997; Talcott *et al*, 1998). More recently these groups have come together in an international collaboration with the publication of the Multinational Association for Supportive Care in Cancer (MASCC) ‘risk index’ (Klastersky *et al*, 2000). In this study more than 40 possible risk factors for the development of significant medical complications at presentation of FN were examined in an initial derivation set of 756 patients. From this, logistic regression analysis was used to derive a model for the risk of development of such complications, which was subsequently tested in a further validation set of patients. The resulting risk index consists of seven weighted clinical factors (Table 1). The value of this scoring system in the prediction of the development of serious complications has subsequently been validated in both the single-centre (Uys *et al*, 2004) and multicentre settings (Paesmans *et al*, 2003).

The aim of defining low-risk neutropenia in this way has been to identify episodes of FN, which may be amenable to newer approaches to treatment involving less intensive, more convenient treatment, which may in turn be expected to have an impact on patients' quality of life. Such treatment strategies may also have a potentially significant impact on health service resource utilisation.

Most research effort has focussed on the role of oral antibiotics. Two large multinational prospective randomised controlled trials (Freifeld *et al*, 1999; Kern *et al*, 1999) have demonstrated equivalence in terms of both efficacy and safety for oral antibiotics compared to standard parenteral regimens for patients with low-risk FN when delivered in the in-patient setting. These findings have led to the inclusion of combination oral antibiotics as a standard option for treatment of low-risk FN in the most recent Infectious Diseases Society of America guidelines for the use of antimicrobial agents in neutropenic patients with cancer (Hughes *et al*, 2002). The case for oral antibiotics has been further strengthened by a recent meta-analysis (Vidal *et al*, 2004) of 15 trials comparing oral and i.v. treatment, which found no difference in failure rates or mortality between the two interventions.

The feasibility of combining an oral antibiotic regimen with early hospital discharge has been reported in a UK population in a randomised single-centre trial (Innes *et al*, 2003) which compared this approach to standard in-patient i.v. treatment. In this study, not only were the success rates of the i.v. and oral arms comparable, but importantly the policy of oral antibiotics with early discharge reduced median in-patient stay to only 2 days. In turn this was associated with substantial savings both in financial terms and in the amount of nursing care required. Although a long-term aim, there is presently little randomised data to support the management of low-risk FN in the outpatient setting.

The evolving management of low-risk FN on an international level led us to investigate the current treatment policy of low-risk FN in oncology departments across the UK. In particular, we were interested to determine:

Whether UK clinicians are assessing the likelihood of patients developing complications related to FN and if so, whether they are using the MASCC index or other means.
Whether UK clinicians are using oral antibiotics as first-line treatment in any patients with FN.
What criteria UK clinicians are using to determine patients' suitability for hospital discharge and whether they have policies for early hospital discharge.

## METHODS

A database of clinicians registered to a UK national trial of prophylactic antibiotics (Cullen *et al*, 2005) was used to identify 249 consultant oncologists and haematologists from 60 centres across the UK with an interest in the antibiotic management of FN. Questionnaires were sent to these identified clinicians in September 2003 with a covering letter explaining its aims and rationale, a stamped addressed envelope and additional questionnaires to distribute to other relevant colleagues.

### The questionnaire

The questionnaire was designed to be succinct (eight questions covering one sheet of paper) and easy to answer consisting of mainly tick-box responses. The name and hospital of the clinician was preprinted onto the questionnaire. Clinicians were initially asked whether they manage patients with FN. If not they were asked to return the survey uncompleted, but otherwise to proceed to answer the remaining questions. The questions related to the three key areas specified below and clinicians were invited to also give further comments.

#### Stratification and treatment of low-risk FN

Clinicians were asked whether they routinely stratify patients with FN and, if so, to explain the criteria by which they defined ‘low-risk’. They were then asked about their standard first-line antibiotic treatment for non-penicillin-allergic low-risk patients with FN. They were asked to specify whether they use an i.v. antibiotic regimen until resolution, an i.v. regimen initially followed by ‘step-down’ oral antibiotics or oral antibiotics from the outset and for the details of the regimen used. There was also an additional question asking about the timing of antibiotic discontinuation.

#### Criteria for hospital discharge

Clinicians were asked for the criteria they use for hospital discharge for patients recovering from febrile netropenia. They were asked whether they take into account temperature, neutrophil count, a combination of both or other criteria. If temperature was used as a criterion for discharge clinicians were asked to further define the absolute value and duration of the patient's defervescence, which would be deemed appropriate for discharge. Similarly, clinicians who specified neutrophil count as a criterion were asked for the absolute neutrophil count which would be deemed appropriate for discharge or alternatively whether they used a rising neutrophil count, irrespective of the absolute value as their preferred criterion.

#### Trial participation

Finally, clinicians were asked whether they would be willing to participate in a trial for low-risk FN using oral antibiotics in conjunction with early hospital discharge.

### Statistical analysis

The responses of clinicians to the questionnaire are reported descriptively. Type of antibiotic treatment and criteria for hospital discharge are compared between those who do or do not stratify patients by risk using continuity-adjusted *χ*^2^ tests.

## RESULTS

Of the 249 questionnaires sent out to clinicians from 60 cancer departments across the UK, replies were received from 114 (47.4%) clinicians who manage patients with FN. A further 14 replies were received from clinicians who were not included in the original mailshot but who had been passed copies of the questionnaire by other recipients. Thus a total of 128 responding clinicians (94 clinical/medical oncologists; 32 haematologists and two others) who manage FN responded. These represent 50 departments across the UK with a median of two clinicians from each centre (range 1–7).

### Stratification of FN

**Figure 1:**
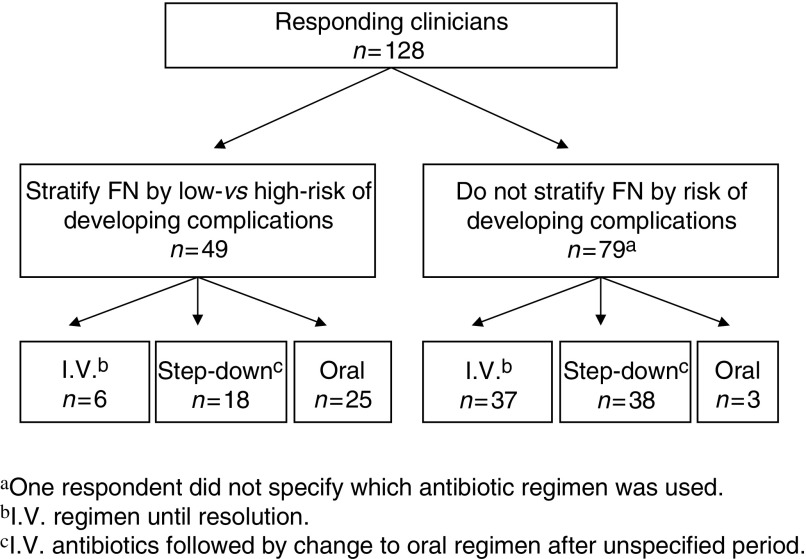
Summary of clinicians' antibiotic treatment of low-risk FN.

Of these 128 clinicians, 79 (62%) do not stratify patients with FN into low- and high-risk categories (Figure 1). Of the 49 (38%) who do stratify, three indicated that this was on an occasional or informal basis. Of the 50 oncology/haematology departments represented, 28 (56%) had at least one clinician who stratifies patients by risk and 22 (44%) had no responding clinician who stratifies such episodes by risk. Within the centres represented there was a lack of consistency regarding risk stratification; of the 29 centres from which two or more clinicians responded, there were only five centres from which all responding clinicians stratify by risk.

Of the 49 clinicians who stratify by risk, 43 gave the criteria by which they define ‘low-risk’, three stated that there were no formal criteria and three gave no further information. The criteria varied substantially, with the majority using a combination of factors including patients' symptoms and signs, absolute neutrophil count and expected duration of neutropenia, nature of the underlying malignancy, previous episodes of neutropenia and age (Table 2). However, very few clinicians appeared to be systematically using a single published definition of low-risk but rather to be using factors derived from more than one study. In addition, some clinicians gave more general and subjective definitions, for example, ‘no sepsis’, ‘well’ etc. Responding clinicians from only one centre (the author's) were routinely incorporating the MASCC risk-index into clinical practice. Clinicians who were using oral antibiotics for low-risk patients also gave patients' ability to tolerate oral medication, for example, not vomiting, able to tolerate oral medication, no severe mucositis as additional criteria for suitability for such treatment. In addition, clinicians who had a policy of early discharge also included additional criteria for their suitability for example, having a carer at home, proximity to hospital, availability of transport, together with subjective assessment of patients, for example, ‘compliant patient’, ‘sensible patient’.

### Antibiotic regimens

Overall 43 (34%) clinicians use an i.v. antibiotic regimen until resolution as their standard first-line treatment for non-penicillin allergic patients with ‘low-risk’ FN, 56 (44%) use i.v. antibiotics followed by oral antibiotics (a ‘step-down’ regimen) and 28 (22%) use an oral antibiotic regimen from the outset in at least some of their patients (Figure 1). Of the 49 clinicians who stratify patients, 25 (51%) use oral regimens from the outset, in marked contrast to the 79 clinicians not stratifying by risk of whom only three (4%) used oral regimens (*P*<0.0001). The range of different antibiotics regimens utilised is shown in Table 3.

### Criteria for hospital discharge

The criteria used by respondents for hospital discharge are shown in Table 4. The majority (*n*=91; 71%) used a combination of temperature and neutrophil count as criteria for hospital discharge (including four who stated that just one criteria is used in certain situations and one whose criteria also included five days i.v. antibiotics). Of the remaining respondents, 33 (26%) used only the patient's temperature, two (2%) used only the neutrophil count, one specified that discharge was at the clinician's discretion and one did not specify. Those who stratify patients according to risk were more likely to utilise fever lysis alone, irrespective of neutrophil count: 17 of the 49 (35%) clinicians who do stratify by risk use temperature only compared with 16 of the 79 (20%) nonstratifying clinicians although this did not reach statistical significance (*P*=0.11). Of the 123 clinicians who specified temperature criteria for discharge, almost half (*n*=60; 49%) specified ⩽37°C and half (*n*=60; 49%) specified ⩽37.5°C, with 24 h the most popular duration (*n*=87; 71%). Of the 93 clinicians who gave patient's neutrophil count as part of their criteria for discharge approximately a third (*n*=32; 34%) use a rising neutrophil count irrespective of the absolute value, while the remainder (*n*=61; 66%) have a fixed absolute neutrophil count which they regard as being suitable for discharge.

### Duration of antibiotic treatment

The majority of clinicians (*n*=79; 62%) stated that in low-risk patients antibiotics were discontinued after a minimum number days following lysis of fever with 1–2 days specified by 14 clinicians, 2–5 days by 54 and 5–7 days by 11. A further 27 (21%) discontinue antibiotics at (or 24 h before; *n*=1) discharge. Nine clinicians (7%) discontinue after a fixed duration from onset of fever with number of days ranging from 5 to 10. The criteria for the remaining clinicians related to neutrophil recovery (*n*=4) or white blood count (*n*=1) and eight not specifying.

### Clinicians' comments and trial participation

When asked for their further comments, clinicians from three centres commented that they manage some patients with low-risk FN as outpatients and a further five that they have early discharge policies in place. A number of clinicians also made comments regarding practical difficulties in the assessment and management of low-risk FN. In particular, several respondents cited difficulties with the ability of junior staff to assess risk, for example ‘junior staff play it safe’; ‘(difficulty in determining whether) the nadir is passed or coming’. One clinician commented that ‘in practice very rarely do patients get into low-risk category’. However, respondents were generally very favourable about the concept of early discharge for patients with low-risk FN, one clinician commenting that ‘we have real pressure on beds, anything reducing in-patient stay would be of interest’. Of the 128 respondents, 108 (84%) said that they would be willing to participate in a trial for low-risk FN of oral antibiotics combined with early hospital discharge.

## DISCUSSION

This study is the first to survey the UK management of FN, an important complication of anticancer chemotherapy. Clinicians from 50 of the 60 cancer departments surveyed (83%) responded, giving broad representation of current treatment across the UK. The findings suggest substantial variation in the management of FN and a slow and/or cautious introduction of recent evidence in this area.

Fewer than half of the responding clinicians stratify patients with FN according to their likelihood of developing complications. Moreover, of those who do stratify, the definition of ‘low-risk’ varies greatly, with clinicians using different combinations of a variety of published and unpublished, objective and subjective criteria. Clinicians from only one centre (the author's) were using the well-validated MASCC scoring system. Moreover, only a quarter (22%) of respondents use oral antibiotics from the outset in the management of any of their patients with FN. This is despite the increasing evidence base, which demonstrates equivalence in terms of success rates and safety for oral *vs* i.v. regimens in low-risk FN (Freifeld *et al*, 1999; Kern *et al*, 1999; Innes *et al*, 2003), the anticipated benefits of quality of life to patients and the potential significant savings in utilisation of resources. Furthermore, the study findings represent the practice of clinical trialists in the UK in the field of FN. It may be expected that as such they are more likely to have incorporated recent study findings into their clinical practice. Therefore, this study may overestimate the use of oral antibiotics in the routine management of FN in the UK. The reasons for not implementing such policies have not been explored in the current survey, but it is possible that some clinicians feel that the level of staffing available is inadequate to safely implement the close monitoring required for such policies.

Interestingly, clinicians and centres that do stratify and thus identify low-risk patients were more likely to have introduced oral antibiotics into their management of FN than those who do not stratify. Thus acknowledgement of the concept of ‘low-risk’ is accompanied by recognition that a ‘one size fits all’ management strategy is not necessarily the most appropriate treatment of FN. Similarly, clinicians who stratified by risk were more likely to use only patients’ temperature rather than temperature together with neutrophil count as criteria for determining discharge from hospital than those who do not stratify by risk. In turn this is likely to reflect shorter durations of hospital admission for stratifying clinicians/centres, although this has not been formally examined.

The published evidence presents a strong case that the introduction of less intensive management strategies could offer significant benefits to patients with low-risk FN in terms of reduced hospitalisation and potentially improved quality of life. In a previous UK study, it was estimated that the approximate costs per episode of low-risk FN treated with oral antibiotics/early discharge were 55% of those for a patient treated as an in-patient treated with i.v. antibiotics (Innes *et al*, 2003). Approximately 70% of all FN episodes are low-risk (Klastersky *et al*, 2000). Thus, if such a management strategy could be safely achieved across the UK, the potential resource savings would be considerable.

There is more than one strategy for using the limited available repertoire of orally bioavailable antibacterials to combat neutropenic infection. Instead of using fluoroquinolones to treat neutropenic infections for patients predicted to be at low risk of complications, they might be used at an earlier time point as prophylaxis. Recent results from a randomised, double-blind, placebo-controlled trial of 1565 patients with solid tumours or lymphoma (Cullen *et al*, 2005) have shown reduction in the frequency of febrile episodes during chemotherapy in patients receiving levofloxacin prophylaxis compared with those receiving placebo (11 *vs* 16%; *P*=0.01). These important results are not in themselves sufficient to recommend a universal prophylaxis strategy because the impact on bacterial resistance patterns has not yet been determined. A targeted treatment approach for actual infections using oral fluoroquinolones might enable less intensive treatment and early hospital discharge and these potential benefits need to be assessed. It is not known whether patients who develop FN despite receiving prophylactic antibiotics could be still be defined as ‘low-risk’ even if they otherwise fulfil the MASCC criteria, since such patients have been excluded from previous studies. Further research in this area is required.

In conclusion, the present study identifies both variations in practice and a degree of caution in implementing evidence-based advances in the management of FN in the UK. Better local and national policies and guidelines are clearly needed. We believe that the introduction of newer strategies may be best achieved in the setting of a well-conducted, multicentre trial. The advent of National Cancer Research Networks across the UK provides a unique opportunity for such a study. A randomised phase III trial comparing standard in-patient management with early hospital discharge in cancer patients receiving oral antibiotics for low-risk FN has been sponsored by Cancer Research UK and will be launched later in the year.

## Figures and Tables

**Table 1:** MASCC Risk index

**Characteristic**	**Score**
*Burden of illness*	
No or mild symptoms^a^	5
Moderate symptoms^a^	3
No hypotension	5
No chronic obstructive pulmonary disease	4
Solid tumour/lymphoma or no previous fungal infection	4
No dehydration	3
Outpatient status at onset of fever	3
Age <60 years	2

a Points attributable to burden of illness are not cumulative.

The maximum theoretical score is therefore 26.

The authors used a threshold of ⩾21 points to define ‘low-risk’.

**Table 2:** Criteria used to define ‘low-risk’

**Factor used to stratify risk of FN**	**No of clinicians using this factor**
Clinical findings	26
Absolute neutrophil count	17
Anticipated duration of neutropenia	16
Patients' symptoms or performance status	12
Underlying malignancy (nonhaematological or nonacute leukaemia)	9
Site of infection	8
Comorbidities	7
Age	5
Chemotherapy regimen	4
Previous fungal infection in haematological malignancy	3
Outpatient at presentation	2
Previous episodes of febrile neutropenia	1
‘Controlled’ cancer	1

**Table 3:** Antibiotic regimens used

**Antibiotic regimen used**	**No. (% of known) clinicians using**
*Oral antibiotic regimens (n*=*28)*	
Ciprofloxacin+co-amoxiclav	18 (69)
Ciprofloxacin	8 (31)
Not specified	2
	
*‘Step-down’ antibiotic regimens (intravenous* → *oral; n*=*56)*	
Dual therapy → oral antibiotics	40 (82)
Monotherapy → oral antibiotics	9 (18)
Not specified	7
	
*Intravenous antibiotic regimens (n*=*43)*	
Dual therapy	25 (68)
Monotherapy	12 (32)
Not specified	6

**Table 4:** Criteria used for patient discharge. (a) Temperature and duration for those who use temperature criterion and (b) Neutrophil count criteria for those who use it

	**1 reading**	**2 readings 4 h apart**	**3 readings 4 h apart**	**24 h**	**Other^a^**	**Total**
(*a*)						
⩽37°C	0	8	1	44	7	60
⩽37.5°C	2	10	3	41	4	60
⩽37.9°C	0	1	0	2	0	3
Total	2	19	4	87	11	123^b^
						
(*b*)						
	**Neutrophil count only**	**Temperature and neutrophil count**	**Other**	**Total**		
⩾0.5 × 10^9^/l	0	36 (and rising in 1)	0	36 (39%)		
⩾1 × 10^9^/l	1	18 (and rising in 3)	3	22 (24%)		
Rising neurophil count irrespective of value	1	29	2	32 (34%)		
Other^c^	0	3	0	3 (3%)		
Total	2	86	5^d^	93		

a Includes 72 h, 1 reading or 24, 24–48, 48, 72, >24 h.

b One respondent who stated temperature and neutrophil count as criteria for patient discharge did not actually specify the criteria.

c Includes 0.8 × 10^9^/l, ⩾0.3 × 10^9^/l, ⩾0.2 × 10^9^/l and rising.

d Two of the clinicians who specified ‘other’ criteria did not use neutrophil count.
